# Brown Adipocyte and Splenocyte Co-Culture Maintains Regulatory T Cell Subset in Intermittent Hypobaric Conditions

**DOI:** 10.1007/s13770-019-00205-y

**Published:** 2019-08-19

**Authors:** Tae Heung Kang, Jung Hwa Park, Donghyeok Shin, Hyungon Choi, Jeenam Kim, Myung Chul Lee

**Affiliations:** 1 Department of Immunology, School of Medicine, Konkuk University, 120-1 Neungdong-ro, Gwangjin-gu, Seoul, 05030 Republic of Korea; 2 Department of Plastic and Reconstructive Surgery, School of Medicine, Konkuk University, 120-1 Neungdong-ro, Gwangjin-gu, Seoul, 05030 Republic of Korea

**Keywords:** Brown adipocyte, Negative pressure, Regulatory T cell, Splenocyte

## Abstract

**Background:**

Brown adipocytes have thermogenic characteristics in neonates and elicit anti-inflammatory responses. We postulated that thermogenic brown adipocytes produce distinctive intercellular effects in a hypobaric state. The purpose of this study is to analyze the correlation between brown adipocyte and regulatory T cell (T_reg_) expression under intermittent hypobaric conditions.

**Methods:**

Brown and white adipocytes were harvested from the interscapular and flank areas of C57BL6 mice, respectively. Adipocytes were cultured with syngeneic splenocytes after isolation and differentiation. Intermittent hypobaric conditions were generated using cyclic negative pressure application for 48 h in both groups of adipocytes. Expression levels of T_regs_ (CD4 + CD25 + Foxp3 + T cells), cytokines [tumor necrosis factor-α (TNF-α) and interleukin-10 (IL-10), and the programmed death-ligand 1 (PD-L1)] co-inhibitory ligand were examined.

**Results:**

Splenocytes, cultured with brown and white adipocytes, exhibited comparable T_reg_ expression in a normobaric state. Under hypobaric conditions, brown adipocytes maintained a subset of T_regs_. However, a decrease in T_regs_ was found in the white adipocyte group. TNF-α levels increased in both groups under hypobaric conditions. In the brown adipocyte group, anti-inflammatory IL-10 expression increased significantly; meanwhile, IL-10 expression decreased in the white adipocyte group. PD-L1 levels increased more significantly in brown adipocytes than in white adipocytes under hypobaric conditions.

**Conclusion:**

Both brown and white adipocytes support T_reg_ expression when they are cultured with splenocytes. Of note, brown adipocytes maintained T_reg_ expression in intermittent hypobaric conditions. Anti-inflammatory cytokines and co-inhibitory ligands mediate the immunomodulatory effects of brown adipocytes under altered atmospheric conditions. Brown adipocytes showed the feasibility as a source of adjustment in physical stresses.

## Introduction

Brown adipose tissue (BAT) is a major site of non-shivering thermogenesis in mammals. The amount of BAT is greater in infants than in adults to counter the effects of hypothermia [1]. In rodents, brown adipocytes cluster in defined anatomical depots that arise from mesenchymal precursor cells derived from the myogenic cell lineage. However, after thermogenic stimulation, brown adipocytes may appear at anatomical sites corresponding to those of white adipose tissue (WAT). This process is entitled the “browning” of WAT [2]. Brown adipocytes that appear in WAT depots differ from those in classical BAT depots and are related to cells derived from the white adipocyte cell lineage [3].

In a recent study, BAT was found to possess a distinctive regulatory T cell (T_reg_) subset that has genetic characteristics distinguishable from other tissues, including serum, inguinal WAT, and spleen. In mRNA sequencing analysis of warm conditioned animals, BAT T_regs_ revealed a group of upregulated genes, namely interleukin 10 (IL-10), chemokine (C-X-C motif) ligand (Cxcl) 1 and 2; which were downregulated in T cells from other tissues [4]. Furthermore, a cluster of genes displayed significant changes in expression in response to a cold challenge, which was determined to be responsible for the cold-specific T_reg_ cell signature in BAT [4, 5]. Inflammatory responses in macrophages co-cultured with brown and white adipocytes are also distinguishable [6]. White adipocytes co-cultured with macrophages exhibit increased gene expression of both pro- and anti-inflammatory genes. In contrast, macrophages co-cultured with brown adipocytes demonstrate either downregulated or unaltered pro-inflammatory gene expression. Brown adipocytes exhibit an intrinsic ability to dampen the inflammatory profile of macrophages, whereas white adipocytes enhance macrophage inflammatory responses.

In this context, the immunologic effects of the BAT depot, which harbors a distinct T_reg_ population that is dominant during the neonatal period, should be studied in response to various inducers and stimuli. However, BAT is fragile and loses its innate characteristics when exposed to certain chemical stimuli [7, 8]. The metabolic function and behavior of brown adipocytes was affected by soft to stiff culture hydrogels, and stiff-porous constructs promoted brown adipogenesis [9]. Therefore, we hypothesized that a physical and mechanical stimulus could support the immunologic effects of BAT [10].

With regard to studies on biophysical aspects of T lymphocytes, T lymphocytes could sense mechanical stiffness and adapt to it. T lymphocytes cultured in a substrate with 100 kPa elastic modulus exhibited efficient migration and pronounced spreading with FOXP3 gene expression, when they had been exposed in physiological stiffness ranging from 0.5 to 100 kPa [11, 12]. Furthermore, researches on physiologic effects of hypobaric condition showed beneficial outcomes on cardiac mitochondria and advanced exercise performance [13, 14]. Hypobaric chamber model has been utilized to validate physical properties of medium and organism studied in it [15, 16]. They presented a diverse series of physiologic responses depending on the intensity and frequency of mechanical stimuli.

In this study, intermittent hypobaric conditions were tested since the thermogenic characteristics of BAT could be a source of adjustment in response to pressure alteration. Brown or white adipocytes were co-cultured with splenocytes, while intermittent hypobaric condition has been applied. Previous researches have exhibited immunomodulatory effects of adipocytes using adipocyte-immune cell interaction models [17, 18]. Additionally, brown adipocytes showed thermogenic characteristics in single cell culture conditions [19].

## Materials and methods

### Experimental animals

Animals were maintained in a pathogen-free mouse facility accredited by the Association for Assessment and Accreditation of Laboratory Animal Care International. All animal protocols were reviewed and approved by the Institutional Animal Care and Use Committee (IACUC) at Konkuk University School of Medicine (Approval No. KU17077). All experiments were performed in accordance with institutional guidelines. Three-week-old male C57BL/6 mice (8–11 g) were obtained commercially from Orient-Bio, Seongnam-si, Gyeonggi-do, Republic of Korea. Forty C57BL/6 mice (n = 40) were utilized for the research. They were housed and fed with irradiated, pelleted food and purified acidified water.

### Brown and white adipocyte culture

The isolation and culture of brown pre-adipocytes were performed as described with modifications [20]. Briefly, a mouse was sacrificed by decapitation, and interscapular depots of brown fat were resected and cut into small pieces. WAT was harvested from the flanks and underwent the same procedures as for BAT. The tissue fragments were shaken in Krebs–Ringer bicarbonate HEPES buffer containing 1 mg/ml collagenase type 1 for 30 min at 37 °C. The digested tissue was filtered through a 100-µm nylon screen. The filtrate was washed twice using 1 × phosphate-buffered saline (PBS). The pellets, which consisted of the stromal-vascular fraction of the tissue, were suspended in “growth medium,” composed of Dulbecco’s Modified Eagle’s Medium (DMEM) F12 supplemented with 10% fetal bovine serum (FBS), 1% penicillin/streptomycin and plated on 24-well suspension plates. After incubation (5% CO_2_, 37 °C) for 7 days to yield confluent pre-adipocytes (designated as “day 0”), differentiation was induced by replacing the media with fresh growth media (induction media) supplemented with 1 μM dexamethasone, 1 μg/ml insulin, 1 μM rosiglitazone, and 1 nM 3,3′,5-triiodo-L-thyronine (T3). On day 3, the medium was replaced with maintenance medium with FBS and supplemented with 1% penicillin/streptomycin, 1 ug/ml insulin, and 1 nM 3,3′,5-triiodo-L-thyronine (T3). Mature brown adipocytes were obtained on day 11 of incubation, since the initiation of differentiation using induction media.

### Co-culture with splenocytes in intermittent hypobaric conditions

Male C57BL/6 mice were euthanized by decapitation, and the spleens were aseptically removed and disrupted by mechanical dissociation. After filtration through a 100-μm nylon screen, splenocytes were resuspended with ACK lysing buffer to remove red blood cells (RBC).

Splenocytes were counted using a Neubauer chamber, centrifuged at 1600 rpm for 3 min, and suspended in culture media RPMI 10 (RPMI 1640 supplemented with 10% fetal bovine serum, 50 units/ml of penicillin/streptomycin, 2 mM l-glutamine, 1 mM sodium pyruvate, and 2 mM non-essential amino acids) at a density of 1 × 10^6^ cells/ml for co-culture.

**Fig. 1:**
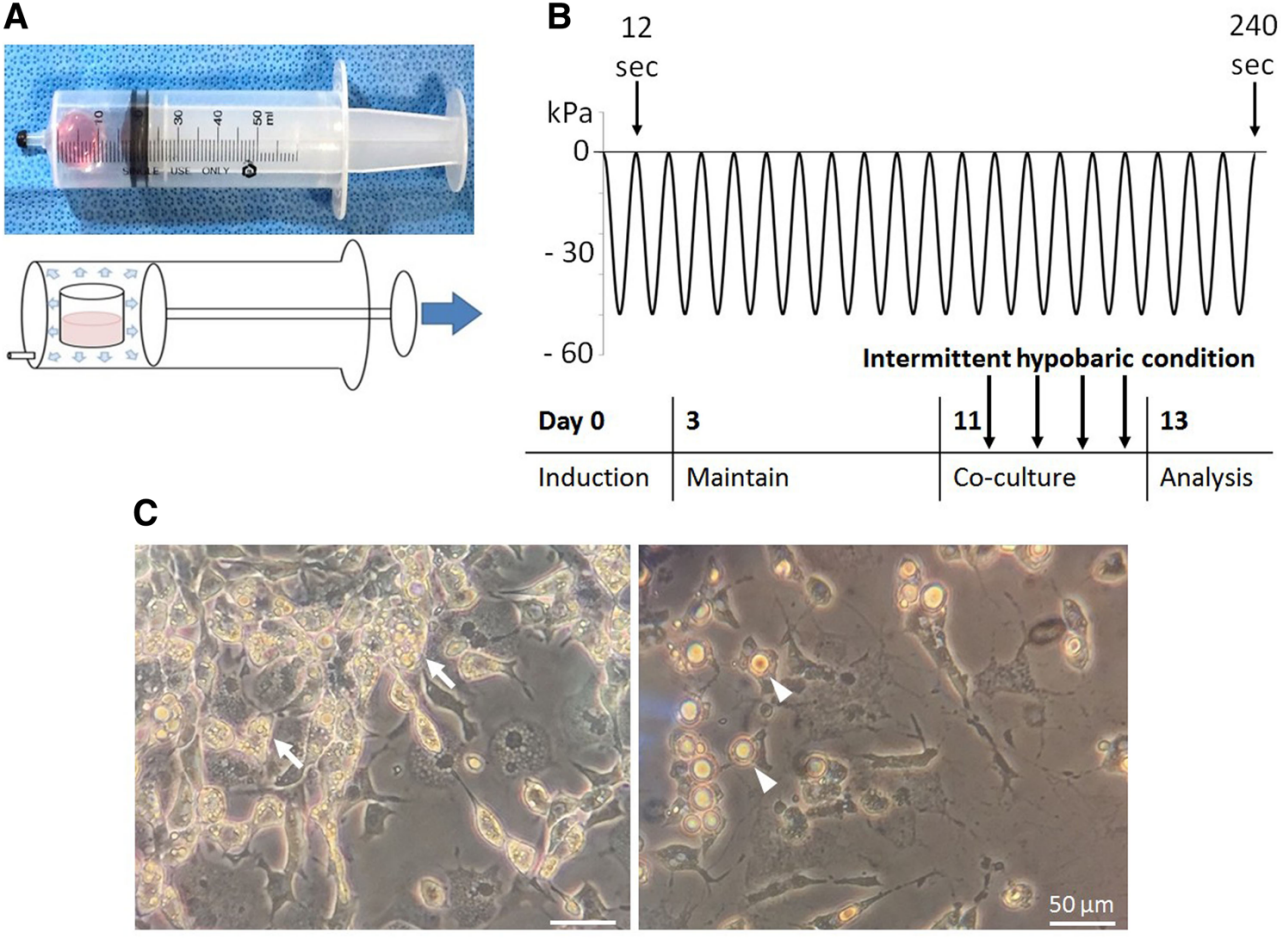
Intermittent hypobaric conditions were generated using cyclic negative pressure application. **A** The medium was maintained in a syringe, and intermittent negative pressure was applied for 48 h using traction on the plunger and barrel. **B** Intermittent hypobaric conditions were present during the co-culture period (days 11–13). Cyclic negative pressure (− 60 kPa) was applied regularly (20 times per set; four sets in a 48-h period). **C** Brown (left) and white (right) adipocytes. Brown adipocytes were identified showing multiple lipid droplets (arrow) under microscopic magnification (× 100). On the other hand, white adipocytes exhibited conventional morphology retaining single lipid droplet (arrowhead) in each cell

To induce intermittent hypobaric conditions, 24-well plates were separated into pieces, and single-well plates were obtained. Separated culture wells were inserted into the barrels of 50-cc syringes, and plunger-barrel assemblies were prepared, each containing a single-well plate (Fig. 1A). Five groups were cultured in intermittent hypobaric conditions; (1) brown adipocytes with splenocytes, (2) white adipocytes with splenocytes, (3) splenocyte mono-culture, and (4) brown or (5) white adipocyte mono-culture groups.

Cyclic negative pressure was applied in the syringe chamber, using traction on the plunger and barrel. Intermittent hypobaric conditions were present during the co-culture period (days 11–13). Negative pressure (− 60 kPa) was applied regularly (20 times per set; four sets in a 48-h period) (Fig. 1B). Intra-chamber pressure was measured using a digital manometer (Rupse HT-1895, Rupse, Hong Kong, People’s Republic of China). Control groups of brown or white adipocytes co-cultured with splenocytes, splenocyte mono-culture and brown or white adipocyte mono-cultures were remained in a resting state during the co-culture. The co-culture process was undergone in culture wells with a single chamber. On day 13, 48 h after splenocyte co-culturing with adipocytes, cell culture media were collected to assess secreted cytokine levels, and splenocytes were harvested as a mixture for flow cytometric analysis.

### Analysis of T_regs_ using fluorescence-activated cell sorting (FACS)

To ascertain the* in vitro* alterations of T cell subpopulations in intermittent hypobaric conditions, we investigated changes in the CD4 + T cell population using flow cytometry. On day 13, splenocytes which had undergone co-culture with brown or white adipocytes were harvested. Flow cytometry was performed using various combinations of fluorochrome-conjugated antibodies to CD4 (RM4-5, eBioscience, San Diego, CA, USA), CD25 (PC61, BioLegend, San Diego, CA, USA), and Foxp3 (FJK-16 s, eBioscience, San Diego, CA, USA). Foxp3 + T cell analysis was performed in accordance with nuclear Foxp3 transcription factor staining standard protocol. Before Foxp3 transcription factor staining, splenocytes were stained with PE-cy7-conjugated anti-CD4 antibodies (GK1.5, eBioscience, San Diego, CA, USA) and APC-conjugated anti-CD25 antibodies (PC61, BioLegend, San Diego, CA, USA) at 4 °C. After 30 min, cells were then washed with PBS and incubated in fixation/permeabilization working solution for 20 min at 4 °C. Finally cells were stained with PE-conjugated anti-Foxp3 antibodies (FJK-16 s, eBioscience, San Diego, CA, USA). Acquired spleen cells were washed and re-suspended in FACS buffer (phosphate-buffered saline, 0.5% bovine serum albumin, 0.1% sodium azide). The stained cells were resuspended in 1 × PBS solution, data were obtained using a FACS Calibur (BD Diagnostic System, Sparks, MD, USA) and analyzed with FlowJo software (TreeStar, San Carlos, CA, USA).

### Determination of cytokine concentration using enzyme-linked immunosorbent assay (ELISA)

The supernatants of adipocytes co-cultured with splenocytes under intermittent hypobaric conditions as well as splenocyte or adipocyte mono-cultures were collected on day 13, and cytokine measurements of tumor necrosis factor-α (TNF-α) and interleukin-10 (IL-10) were performed using an enzyme-linked immunosorbent assay (ELISA), according to the manufacturer’s protocol. ELISA plates were coated with 100 µl/well of capture antibody and incubated overnight at 4 °C. Aspiration and washing were performed three times with 250 µl/well wash buffer. To prevent nonspecific enzyme binding, 1x ELISA/ELISPOT diluent buffer was added for blocking method at RT (real-time) temperature for 1 h. After washing, all samples acquired from cell culture were incubated at RT temperature for 2 h. Standards were diluted to prepare the top concentration and incubated at the same time. After sample incubation, the plate was washed 3 times and detection antibody diluted in 1x ELISA/ELISPOT diluent was added. The plate was sealed and incubated at room temperature for 1 h. After aspiration and washing, Avidin-HRP diluted in 1x ELISA/ELISPOT diluent was added. The plate was sealed and incubated at room temperature for 30 min. Aspiration and washing were followed by adding 50 µl of stop solution to each well. The plate was read at 450 nm.

### Analysis of PD-L1 expression in brown adipocytes using flow cytometry

Mature brown and white adipocyte mono-cultures were collected for the analysis of programmed death-ligand 1 (PD-L1) expression. Adipocytes underwent flow cytometry at day 13, when they had been cultured under intermittent hypobaric condition after maturation. Cells were stained for 30 min at 4 °C with PE-conjugated anti-PD-L1 antibodies (10 F.9G2, BioLegend, San Diego, CA, USA), washed with 1x PBS solution, and resuspended after centrifugation. Both fractions were analyzed by flow cytometry.

### Statistical analysis

Continuous variables were expressed as the mean ± standard deviation (SD) or median [interquartile range (IQR)]. The t test was used to analyze ELISA outcomes. Non-parametric Mann–Whitney U test was used to evaluate T_regs_ and PD-L1 expression. A *p* value less than 0.05 was considered statistically significant. Statistical analyses were performed using SPSS, version 22 (SPSS Inc., Chicago, IL, USA).

## Results

### Brown and white adipocyte differentiation and CD4 + T cell expression

The isolation and differentiation (7 days) of brown and white pre-adipocytes have been followed by induction (3 days) and maintenance (8 days) process. Under microscopy, brown adipocytes possessed multiple lipid droplets. On the other hand, white adipocytes exhibited a conventional morphology with a single lipid droplet present in each cell (Fig. 1C). During differentiation, both brown and white adipocytes demonstrated an increase in their cell numbers and altered intracellular components. The co-culture of splenocytes with adipocyte groups, namely brown or white adipocytes was initiated at the end of maintenance period. The intermittent hypobaric condition was generated using cyclic negative pressure application during the 48 h co-culture period (Fig. 1B).

**Fig. 2:**
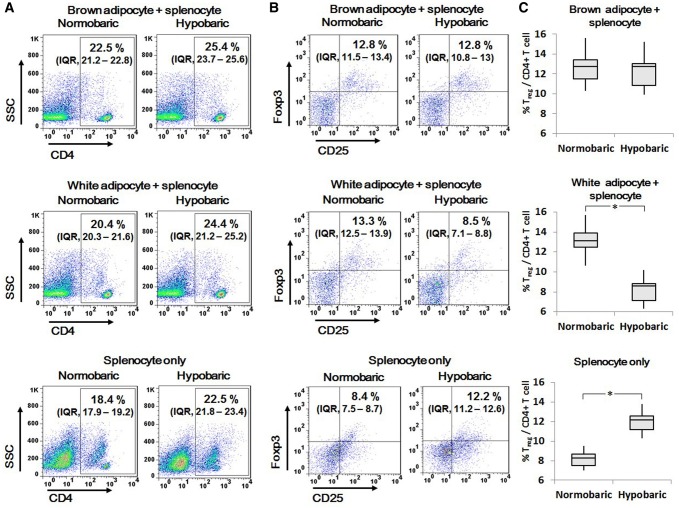
CD4 + T cell and T_reg_ expression under intermittent hypobaric conditions. **A** Brown and white adipocyte groups exhibited comparable CD4 + T cell expression. Intermittent hypobaric conditions resulted in slight increment of CD4 + T cells, however statistical significance was not noted. **B**, **C** Brown and white adipocyte groups exhibited comparable T_reg_ expression under normobaric conditions [brown adipocyte-splenocyte 12.8% (IQR, 11.5–13.4%); white adipocyte-splenocyte 13.3% IQR, 12.5–13.9%)]. The splenocyte only group presented less T_reg_ compared to co-culture groups [8.4% (IQR, 7.5–8.7%), *p *< 0.05]. In response to intermittent hypobaric conditions, the brown adipocyte group maintained a similar level of T_reg_ expression to that in the normobaric state [12.8 to 12.8% (IQR, 10.8–13%)]. However, the white adipocyte group demonstrated a decrease in its T_reg_ population [13.3 to 8.5% (IQR, 7.1–8.8%), *p *< 0.05]. The splenocyte only group exhibited increased T_reg_ in hypobaric conditions [8.4 to 12.2% (IQR, 11.2–12.6%), *p *< 0.05]. **C** The Y axes of bar graphs represent the percentage of T_reg_ among CD4 + T cells

When splenocytes were co-cultured with brown and white adipocytes under normobaric conditions, both groups exhibited comparable CD4 + T cell expression in fluorescence-activated cell sorting (FACS) analysis (Fig. 2A). In response to intermittent hypobaric conditions, both groups showed slight increment in CD4 + T cell, however statistical significance was not noted.

### The effect of intermittent hypobaric condition on regulatory T cell population

With regard to T_reg_ analysis, the brown and white adipocyte co-culture groups exhibited comparable T_reg_ expression under normobaric conditions [brown adipocyte-splenocyte 12.8% (IQR, 11.5–13.4%); white adipocyte-splenocyte 13.3% (IQR, 12.5–13.9%)]. The control splenocyte only group presented smaller T_reg_ ratio in comparison with co-culture groups [8.4% (IQR, 7.5–8.7%), *p *< 0.05].

In hypobaric conditions, the brown adipocyte group retained a similar level of T_reg_ expression to that in the normobaric state [12.8 to 12.8% (IQR, 10.8–13%)]. However, the white adipocyte group exhibited a decrease in its T_reg_ population [13.3 to 8.5% (IQR, 7.1–8.8%), *p *< 0.05]. On the other hand, the splenocyte only group showed T_reg_ increment in hypobaric conditions [8.4 to 12.2% (IQR, 11.2–12.6%), *p *< 0.05] (Fig. 2B, C). Differences among the groups demonstrate distinct intercellular responses between brown adipocytes and splenocytes.

### Analysis of cytokine level depending on the intermittent hypobaric condition

**Fig. 3:**
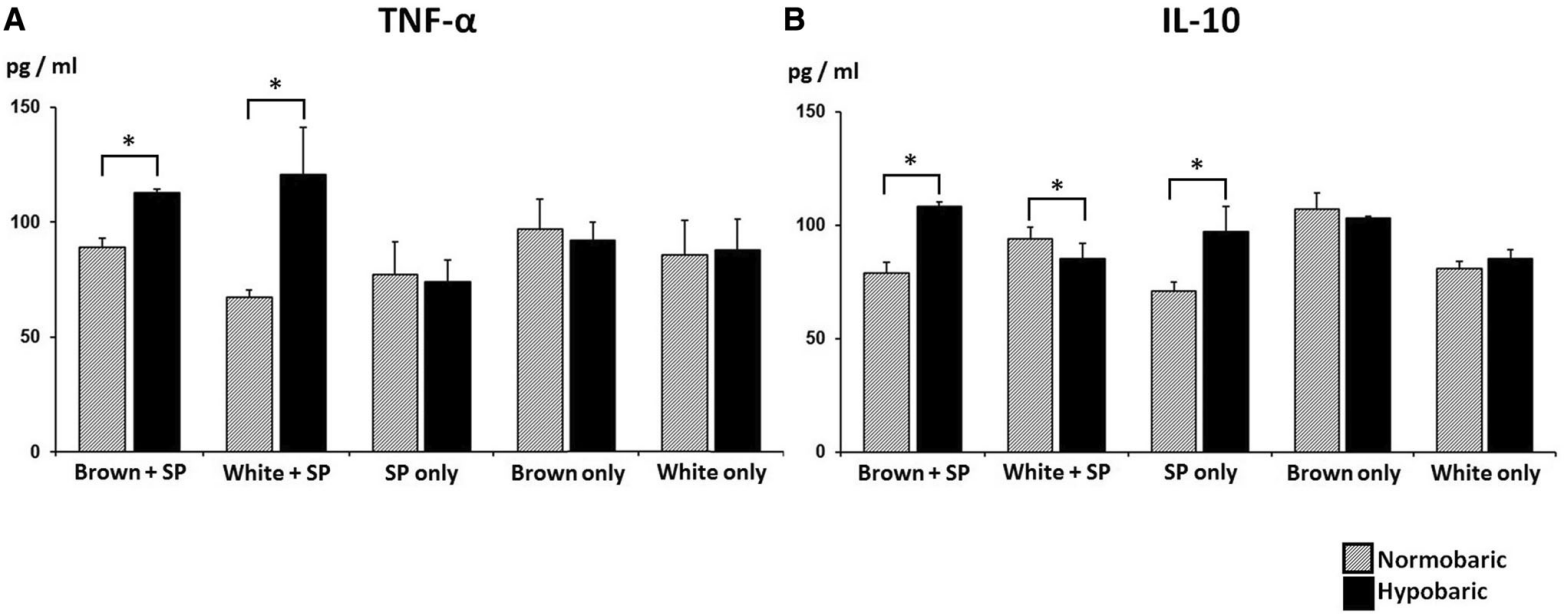
Measurement of TNF-α and IL-10 under intermittent hypobaric conditions. **A** Both brown and white adipocyte co-culture groups demonstrated increased TNF-α level under intermittent hypobaric conditions (*p *< 0.05). However, the splenocyte only group did not exhibit a significant change. **B** The level of the anti-inflammatory factor IL-10 was increased significantly in brown adipocyte co-culture and splenocyte only mono-culture groups (*p *< 0.05). Meanwhile, exposure to hypobaric conditions led to a decrease in the level of IL-10 in white adipocyte co-culture group. Brown adipocyte mono-culture group presented more IL-10 in comparison with white adipocyte mono-culture group at base line. (Brown + SP, brown adipocyte and splenocyte co-culture; White + SP, white adipocyte and splenocyte co-culture; SP only, splenocyte only mono-culture; Brown only, brown adipocyte only mono-culture; White only, white adipocyte only mono-culture)

With regard to cytokine analysis related to intercellular responses, the brown and white adipocyte co-culture groups possessed increased TNF-α levels under intermittent hypobaric conditions (brown adipocyte co-culture group, 89 ± 3.9 to 113 ± 1.6 pg/ml; white adipocyte co-culture group, 67 ± 3.1 to 121 ± 20.4 pg/ml; *p *< 0.05). In control groups, the splenocyte only mono-culture group did not show significant alteration (77 ± 14 to 74 ± 9 pg/ml). Brown and white adipocyte mono-culture groups also presented comparable TNF-α levels with regard to intermittent hypobaric conditions (brown adipocyte mono-culture group, 97 ± 13 to 92 ± 8 pg/ml; white adipocyte mono-culture group, 86 ± 15 to 88 ± 13 pg/ml) (Fig. 3A).

The anti-inflammatory factor IL-10 level presented a distinguishable outcome in intermittent hypobaric conditions. The brown adipocyte co-culture group exhibited a significant increase in IL-10 level (79 ± 4.5 to 108 ± 2 pg/ml, *p *< 0.05). Meanwhile, exposure to hypobaric conditions led to decreased IL-10 in the white adipocyte co-culture group (94 ± 5.1 to 85 ± 7.1 pg/ml, *p *< 0.05). The control splenocyte only group presented IL-10 increment (71 ± 4 to 97 ± 11 pg/ml, *p *< 0.05). Brown and white adipocyte mono-culture groups did not show significant alteration with regard to intermittent hypobaric conditions (brown adipocyte mono-culture group, 107 ± 7 to 103 ± 1 pg/ml; white adipocyte mono-culture group, 81 ± 3 to 85 ± 4 pg/ml). Nonetheless, brown adipocyte mono-culture group exhibited greater IL-10 level than white adipocyte group at base line (107 ± 7 vs 81 ± 3 pg/ml, *p *< 0.05) (Fig. 3B).

Brown adipocytes demonstrated an ability to balance the expression of pro- and anti-inflammatory cytokines, whereas white adipocytes had higher pro-inflammatory cytokine level in the hypobaric state.

### The effect of intermittent hypobaric condition on PD-L1 expression

**Fig. 4:**
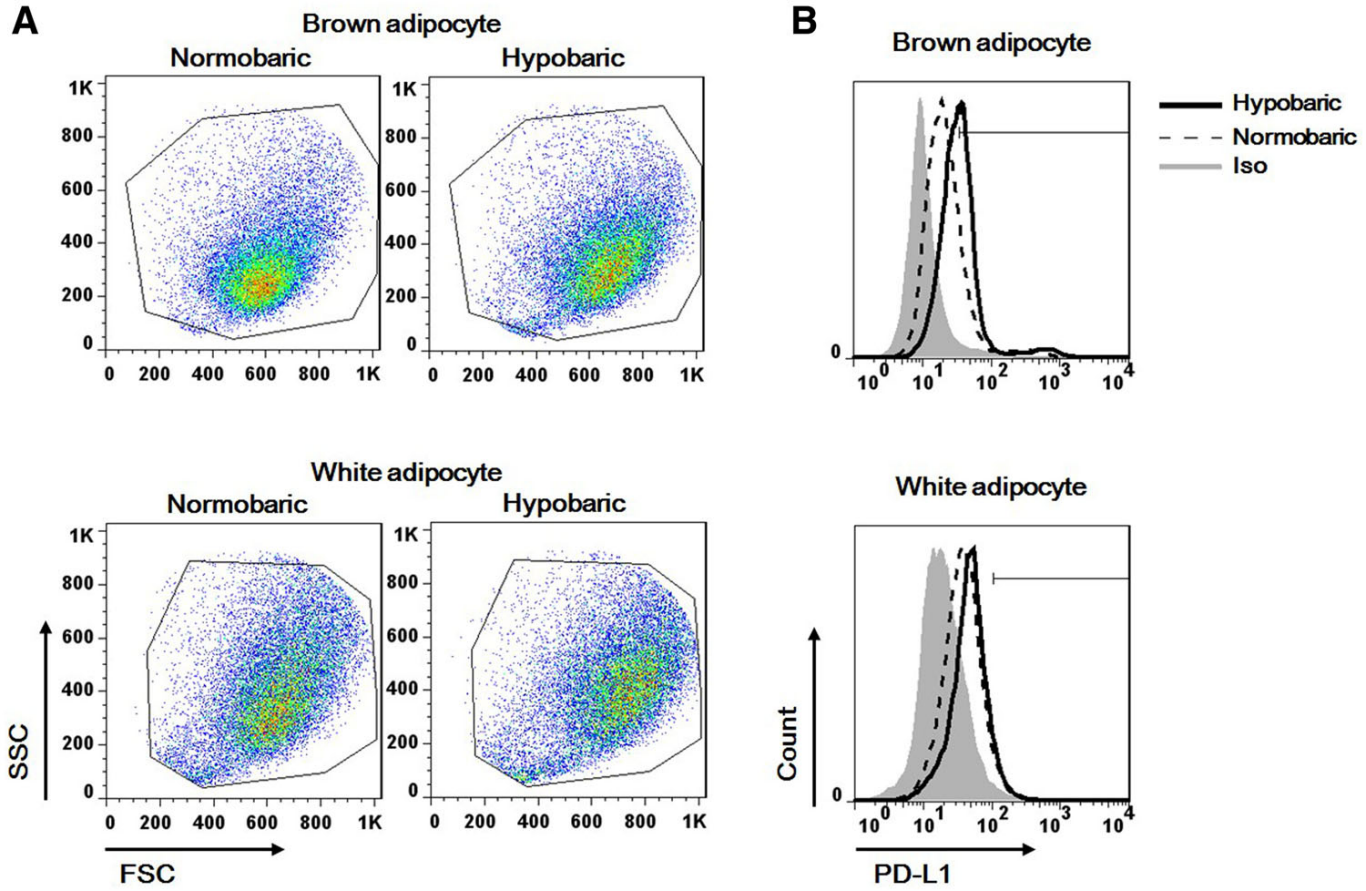
Analysis of PD-L1 expression using flow cytometry in **A** dot plots and **B** histogram. Brown adipocytes expressed significantly higher levels of PD-L1, 19.0% (IQR, 17.8–19.3%) than white adipocytes, 5% (IQR, 4.2–6.3%) under normobaric conditions (*p *< 0.05). In addition, intermittent hypobaric condition exerted more prominent increment in brown adipocyte group [19.0 to 46.5% (IQR, 42.1–47.8%)], compared to white adipocyte group [5 to 7.4% (IQR, 6.5–7.9%)] (*p *< 0.05)

PD-L1 expression was also analyzed; PD-L1 was determined to be an activation independent marker of brown adipocyte [21]. In PD-L1 quantification using FACS analysis, brown adipocyte group showed notable expression, 19.0% (IQR, 17.8–19.3%) compared to white adipocyte group, 5% (IQR, 4.2–6.3%) at normobaric condition (*p *< 0.05). Furthermore, hypobaric condition resulted in more significant increment in brown adipocyte group [19.0 to 46.5% (IQR, 42.1–47.8%)] than white adipocyte group [5 to 7.4% (IQR, 6.5–7.9%)] (*p *< 0.05) (Fig. 4).

## Discussion

T_regs_ represent a subpopulation of CD4 + T cells that specifically express Foxp3. In recent studies, the potent and stable expression of Foxp3 has been regarded as a key element in T_reg_ effectiveness [22]. The process by which T_regs_ are generated depends on the expression levels of Foxp3, which do not fluctuate under conditions of T cell activation [22, 23]. Identifying adequate stimuli at appropriate levels to induce stable Foxp3 expression with effective T_reg_ function is important for the investigation of immunomodulation.

T_regs_ play roles in crucial defense mechanisms against inappropriate immune responses and compose 5–20% of the CD4 + compartment. They operate during conditions of inflammation, infection, allergy, autoimmunity, and tumorigenesis [18]. T_regs_ exist at high levels in human cord blood (12% of all CD4 + T cells) and neonatal lymph nodes (8%). Naive neonatal T cells demonstrate a propensity to differentiate into T_regs_ in response to maternal antigens that cross the placenta [24, 25]. Human undifferentiated neonatal T cells (CD4 + CD8–Foxp3–) have an innate switching mechanism to differentiate into CD4 + Foxp3 + T_regs_ to exert suppressive roles. T_regs_ are crucial determinants in the control of immune responses and metabolic processes; they interact with the innate and adaptive immune system, serving as negative-feedback regulators and ensuring self-tolerance [4, 26].

Applying intermittent low-grade hypobaric pressure (− 60 kPa) has led to T_reg_ maintenance via brown adipocyte co-culture. However, the change in pressure resulted in a failure of T_reg_ maintenance when white adipocytes were located adjacent to splenocytes. Various cellular responses depending on culture condition have been studied using lymphocytes and adipocytes [12, 27]. Actomyosin mediated tension has been suggested as the key factor of uncoupled respiration in brown adipocytes. Intracellular calcium influx and myosin light chain kinase (MYLK) activity have generated tension on the cytoskeleton and led to increased cellular elasticity [28]. Mechanical stimuli and environmental alteration resulted in distinctive cytologic expression and differentiation in previous researches [29, 30].

Brown adipocytes function in the homeostasis of energy expenditures. BAT thermogenesis is linked to the expression of the thermogenic factors such as, uncoupling protein 1 (UCP1) and type II deiodinase [31]. The transporter UCP1 upsets the mitochondrial proton gradient, thereby converting the energy produced by the mitochondrial oxidation of fatty acids from ATP formation into heat. BAT responses to cold stimuli and the “browning” of WAT have been shown to involve immune cells, including macrophages. Adult humans have functional BAT, which plays a role in energy balance [32].

Furthermore, the homeostatic capacity of BAT can be observed under different environmental conditions, including temperature [33]. In a recent study, cold adaptation led to elevated expression levels of genes involved in stress pathway of BAT endoplasmic reticulum (ER)-localized transcription factor (nuclear factor erythroid 2–like 1, Nrf1). Low temperature resulted in BAT gene expression and the regulation of respiratory capacity. Brown adipocytes are stimulated via β3-adrenergic receptor upon cold exposure [34]. The expression mechanism of brown adipocytes in contractile signaling strongly imitates the process of cardiomyocytes; involving cAMP, PKA, L-type Ca^2+^ channel, and MYLK cascade [28].

In this respect, adrenergic receptor related mechanical stimuli, such as the alteration of atmosphere or viscoelasticity can be considered to validate mechano-sensitive brown adipocyte effects [28, 35]. The control of cellular activation, proliferation and differentiation using mechanoregulation has been presented via internally developed or externally applied physical stimuli [36, 37].

Mitochondria and the endoplasmic reticulum are in close proximity in brown adipocytes, with lipid droplets occupying the majority of the intracellular space. Recent evidence suggests that endoplasmic reticulum membranes are fused to the outer mitochondrial membrane in BAT, providing an opportunity for communication between these organelles. Such histology facilitates adaptability while reducing the potential for interference from radical environmental changes [19].

In a recent study, BAT was shown to possess a unique subset of T_regs_ characterized by a unique gene signature. After exposure to cold, these T_regs_ responded to BAT activation, and the systemic ablation of T_regs_ compromised the adaptation of whole-body energy expenditure to the cold, consistent with impairment in thermogenic marker gene expression and the massive invasion of pro-inflammatory macrophages into BAT [4].

Brown adipocyte and macrophage co-culture using immortalized brown and white adipocytes exhibited distinguishable levels of gene expression [6]. IL-6, which is involved in chronic inflammation, was present at lower levels in brown adipocytes than in white adipocytes. White adipocytes had a lower threshold of activation in response to inflammatory signals, such as extrinsic and environmental factors.

We analyzed the levels of TNF-α and IL-10, and found that intermittent hypobaric conditions caused different levels of secreted factors between brown and white adipocyte groups. Brown adipocyte group secreted higher levels of IL-10 than white adipocyte group, demonstrating adaptive capability to environmental changes, namely intermittent low-grade hypobaric conditions. In a research, IL-10 was expressed dominantly in brown adipocytes of warm conditioned animals, although brown adipocytes have been typically stimulated in cold temperature [4]. The tolerable range to secrete anti-inflammatory cytokine, such as IL-10 is considered to be diverse in brown adipocytes [38].

On the other hand, levels of TNF-α increased in both groups. TNF-α has been presented as a key molecule, involved in adipose tissue browning and energy homeostasis of nephropathic mouse model [39]. A series of molecules, including cytochrome c oxidase subunit II (COX2), prostaglandin F2α (PGF2α), interleukin 1α (IL-1α), interleukin 6 (IL-6), tumor necrosis factor α (TNF-α) was expressed dominantly, showing increased energy expenditure. The intermittent hypobaric condition could be considered both an inflammatory stress and an anti-inflammatory stimulus in brown adipocytes.

In brown adipocyte and splenocyte co-culture group, the balance between TNF-α and IL-10 alteration could lead to the maintenance of T_reg_ level. Meanwhile, white adipocyte and splenocyte co-culture group presented increased TNF-α and decreased IL-10 with regard to intermittent hypobaric condition. TNF-α expression with anti-inflammatory cytokine IL-10 decrement could explain the failure of T_reg_ maintenance. Further research to reveal the interrelation between adipocytes, inflammatory cytokines, and energy homeostasis is necessary in various culture conditions.

Allogeneic adipose-derived stem cells have been used as a model of immune regulatory effects. Mesenchymal stem cell-mediated suppression of T cell proliferation occurs via the upregulation of PD-L1 [40]. PD-L1 signal on dendritic cells is critical for the induction of T_reg_ tolerance in livers transplanted into mice [41]. The effects of dendritic cells on T_reg_ induction and expansion appear to depend on PD-L1. The alteration of PD-L1 level has been studied using lung cancer cells, and they responded to matrix stiffness [30]. Substrates with greater elastic modulus (25 kPa) resulted in higher PD-L1 expression than substrates with lower modulus (2 kPa). Actin-dependent signaling was noted as the mechanism of PD-L1 alteration. Intermittent hypobaric condition caused significant increase of PD-L1 expression in brown adipocytes. Brown adipocytes also respond to the matrix microenvironment via actomyosin cytoskeleton mediated pathways [28]. The innate characteristics of brown adipocytes delineate their immunomodulatory capacity.

The control splenocyte mono-culture group demonstrated less T_reg_ expression in the normobaric condition, compared to two adipocyte co-culture groups. Nonetheless, intermittent hypobaric condition has led to an increase in T_reg_ population, and the range was comparable to brown or white adipocyte and splenocyte co-culture groups in normobaric condition. In terms of cytokine analysis, splenocyte mono-culture resulted in IL-10 increment without change in TNF-α level, meanwhile adipocyte and splenocyte co-culture groups exhibited alteration of both IL-10 and TNF-α in hypobaric conditions. Independent mechanical signaling of T lymphocyte delineates the outcome [11, 26]. T cell receptors can both sense a force and convert it into biochemical signals. Previous studies have exhibited diverse activation range of T lymphocyte depending on the culture condition with physiologic stiffness profile [42, 43]. Soft viscoelastic modulus was measured in primary CD4 + T cell, whereas greater elastic modulus was shown in antigen presenting cells in inflammatory conditions.

Brown or white adipocyte co-culture with splenocytes has assisted the maintenance or alteration of T_reg_ population. The balance among immunomodulatory molecules including IL-10, TNF-α and PD-L1 played roles in T_reg_ maintenance effect mediated by brown adipocytes.

Brown adipocytes have presented fragility, when they received certain chemical stimuli [7, 8]. In our research, intermittent low-grade hypobaric pressure was considered, since thermogenic characteristic and the close interaction between mitochondria and endoplasmic reticulum could be a source of adjustment in pressure alteration [19, 33]. Both adipocytes and splenocytes endured low-grade hypobaric condition, retaining cellular integrity.

Among various mechanical modalities, stretching stimuli have been utilized in both adipocytes and immune cells. Static stretching of adipocytes resulted in the enhancement of adipogenesis via Rho/Rho kinase pathway; however cyclic stretching showed the inhibition of adipogenesis via MEK 1/2, Smad2 and β-catenin pathways [44, 45]. Furthermore, cyclic stretching suppressed IL-1β secretion by attenuating the AMP kinase pathway in macrophages. Cyclic stretching is regarded as homeostatic condition preventing excessive inflammasome activation [46, 47]. Both hypobaric and stretching condition showed various outcomes in homeostasis regulating pro- and anti-inflammatory pathways.

There is a limitation of our study, since accurate temperature measurement related to interaction between brown adipocytes and pressure alteration has not been undergone. In a recent research, the thermogenic characteristic of brown adipocyte was presented using a small molecule-type thermosensitive fluorescent dye, and fluorescence intensity has shifted in response to adrenergic stimuli, which corresponded to temperature alteration [19]. Another research demonstrated that brown adipocytes have utilized mechanosensitive transcriptional co-activators, and actomyosin-mediated elasticity regulated thermogenic capacity of adipocytes [28]. The dynamic response of brown adipocytes in various culture conditions should be studied in further researches.

The neonatal immune system is differentiated from the adult immune system in terms of its propensity for tolerance [48]. Neonatal and adult immune responses against common bacterial organisms can thus be distinguished, since the population of commensal-specific CD4 + T cells is dominated by T_regs_ in neonates. Meanwhile, effector T cells are more plentiful than T_regs_ in adults. Mechanisms to promote immune tolerance to commensal bacteria are preferentially active during the neonatal period. Of note, brown adipocytes are present in higher numbers during the neonatal period than in adults. In this context, the immunomodulatory capacity of brown adipocytes should be studied, while preserving their innate characteristics.
